# Age, gender and voided volume dependent nomograms

**DOI:** 10.4103/0970-1591.70597

**Published:** 2010

**Authors:** Arvind P. Ganpule

**Affiliations:** Consultant Urologist and Chief, Division of Laparoscopy, Nadiad, Gujarat, India

Dear Editor,

We read with great interest the article by Vikashkumar *et al*.[1] We commend the authors for their work in depicting age, gender and volume-dependent “nomograms” in a population subset. We would also like to draw the attention of the authors to a few points and make a few comments.

First, although the authors state that the “volunteers” were healthy, one cannot be sure that how on just eliciting the history, the volunteer is “deemed” free of a urological disease. A baseline ultrasound examination and urine examination would have been more prudent to label these “volunteers ”as healthy.

Second, it is not clear how the authors have calculated the Qmax. The ideal method would be a sustained peak on the tracing which is maintained for at least two seconds. This assumes importance as sometimes artifacts and an isolated peak on the tracing tends to give false readings as regards the value.

Third, the title of the study mentioning “Indian population” is inappropriate and misleading for the readers. India being a socioculturally and ethnically diverse country, the data and these results cannot be extrapolated for the Indian population as such, further they cannot definitely be representative of the population. If the study would had been a well-designed multicenter “community”-based study with a statistically significant and appropriate sample size across a cross-section of the population such a nomenclature would have been appropriate.

Fourth, on close analysis of the data there appears to be inconsistency and confusion regarding the number of “healthy volunteers”. On calculating the numbers as given in the article, the number of “healthy volunteers” is 1017 (not 1011 as mentioned by the authors) (page 462). There also appears to be a discrepancy in the number of premenopausal and post-menopausal females. The authors have stated the number of premenopausal females as n=207 and once as 202 on the same page! (page 462). To say the least, such errors are not acceptable in a paper selected as a “prize paper”!!!

We would like to wish to bring to the notice of the author that a study has been published in India with similar conclusions.[2]
